# Gene Expression Pattern in Olive Tree Organs (*Olea europaea* L.)

**DOI:** 10.3390/genes11050544

**Published:** 2020-05-12

**Authors:** Jorge A. Ramírez-Tejero, Jaime Jiménez-Ruiz, María de la O Leyva-Pérez, Juan Bautista Barroso, Francisco Luque

**Affiliations:** Center for Advanced Studies in Olive Grove and Olive Oils, Department of Experimental Biology, University Jaén, 23071 Jaén, Spain; jrtejero@ujaen.es (J.A.R.-T.); ruiz@ujaen.es (J.J.-R.); Maria.Leyva@teagasc.ie (M.d.l.O.L.-P.); jbarroso@ujaen.es (J.B.B.)

**Keywords:** RNA-seq, plant organs, transcriptome, housekeeping genes

## Abstract

The olive tree (*Olea europaea* L.) was one of the first plant species in history to be domesticated. Throughout olive domestication, gene expression has undergone drastic changes that may affect tissue/organ-specific genes. This is an RNA-seq study of the transcriptomic activity of different tissues/organs from adult olive tree cv. “Picual” under field conditions. This analysis unveiled 53,456 genes with expression in at least one tissue, 32,030 of which were expressed in all organs and 19,575 were found to be potential housekeeping genes. In addition, the specific expression pattern in each plant part was studied. The flower was clearly the organ with the most exclusively expressed genes, 3529, many of which were involved in reproduction. Many of these organ-specific genes are generally involved in regulatory activities and have a nuclear protein localization, except for leaves, where there are also many genes with a plastid localization. This was also observed in stems to a lesser extent. Moreover, pathogen defense and immunity pathways were highly represented in roots. These data show a complex pattern of gene expression in different organs, and provide relevant data about housekeeping and organ-specific genes in cultivated olive.

## 1. Introduction

The olive tree (*Olea europaea* L.) was one of the first plant species to be domesticated in history. The demand of olive oil and extra virgin olive oil (EVOO), which is the main product obtained from this crop, is continuously increasing, and its health benefits are well-established. Among the different varieties, the EVOO from cultivar “Picual” has exceptional organoleptic properties and marked oxidative stability due to its high content in polyphenolic compounds [1,2]. “Picual” is one of the most important olive varieties worldwide and represents 50% of all the olives trees in Spain, and approximately 20% in the world [3]. However, olive tree breeding is a process that requires decades and has been limited by the genetic knowledge about this plant species being scarce until very recently. In fact, understanding the molecular basis of agronomic traits would allow improved efficiency of breeding programs. The development of genomic information and tools for breeders should now be possible because the genomes of wild olive trees (*Olea europaea* var. *sylvestris*) and cultivars “Farga” and “Picual” have been assembled in the last 3 years [4,5,6]. In addition, 50 more genotypes, 40 cultivated and 10 wild trees, have been sequenced [6]. The “Picual” genome turned out to be more complex than previously reported genomes. Most genes appear to have several paralogs, including genes that are unique in *Arabidopsis*. Knowing whether all the paralog genes are expressed or simply one or a few of them, or if they present a differential expression pattern depending on the plant organ or only in response to stimuli, may be relevant to understand the role of each paralog. Therefore, obtaining an atlas with the gene expression profile in main olive plant organs might be extremely useful to further understand olive tree metabolism and to provide future breeding genetic markers. Tissue-/organ-specific genes confer each plant tissue/organ differential characteristics. Very recently, tissue-specific variations in gene expression and metabolites have been described in fruit, as well as in young and old leaves, in olive [7]. During olive domestication, gene expression has undergone drastic changes [8] that may affect tissue-/organ-specific genes. In the present work, the specific transcriptomic profile in this crop’s main organs is reported using “Picual” as the model cultivar for its relevance for both the scientific community and breeders who seek higher productivity and olive oil quality. Together with genomic data, previous transcriptomic studies of olive tree in response to stresses [9,10,11,12], during plant development [13,14,15,16] or across different plant organs, represent valuable information in the search for the key genes involved in different relevant traits for agronomical purposes.

## 2. Materials and Methods

### 2.1. Plant Material, RNA Sample Preparation, and Next-Generation Sequencing 

The plant samples employed for this study were obtained from the World Olive Germplasm Collection (WOGC) of the Andalusian Institute of Agricultural and Fisheries Research and Training (IFAPA) [17]. Samples were collected from roots, stems, meristems, leaves, flowers, and fruits (epicarp and mesocarp) of three healthy 10-year-old olive trees under field conditions. All the samples were collected at the same time, except for fruits, which had to be collected later upon reaching the turning ripening state. All the samples were immediately frozen in liquid nitrogen and kept at −80 °C until total RNA extraction. For RNA isolation, the Spectrum Plant Total RNA kit (Sigma-Aldrich, St. Louis, MO, USA) was used following the manufacturer’s instructions. Any DNA contamination was removed by a column DNase I treatment step (Roche, Basel, Switzerland). RNA quality tests were performed with the Agilent 2100 Bioanalyzer (Agilent Technologies, Santa Clara, CA, USA) employing an RNA 6000 Pico assay kit (Agilent Technologies), and all the samples’ RNA integrity number (RIN) values ranged from 6.00 to 9.10. Next generation sequencing (NGS) was performed by Sistemas Genómicos (Valencia, Spain). The pool of complementary DNA (cDNA) libraries was sequenced by paired-end sequencing (100 x 2) with an Illumina HiSeq 2000 sequencer. Two biological replicates per sample were sequenced, and each biological replicate consisted in an equilibrated pool of three plant RNAs. Each replicate was loaded twice on two different lanes in the flow cell. 

### 2.2. Data Preprocessing

The raw Illumina RNA-seq reads were first preprocessed using FastqMcf [18] by discarding the primers and reads with adaptors, unknown nucleotides, and poor-quality or short-length reads, which increased the Q score to more than 30 for all the libraries and filtering for lengths longer than 50 bp (Q30L50). Sequencing quality control was performed with the NGS QC software [19]. The trimming and cleaning of the obtained reads were carried out by the Trim Galore software (https://github.com/FelixKrueger/TrimGalore). 

### 2.3. Expression Analysis

The gene expression study was conducted with the DNAStar (ArrayStar 15, Rockville, MD, USA) Qseq software for RNA-seq analyses (www.dnastar.com). Reads were mapped to the *Olea europaea* cv. “Picual” genome as the reference Oleur061 [6]. Mapping was undertaken using parameters k-mer = 63 and 95% of matches. The default normalization method of reads per kilobase per million mapped reads (RPKM) was employed. A basal expression level of log_2_ RPKM = -2 was considered. Therefore, only those genes with expression values above this threshold level were considered expressed, whereas those with values that equaled or were below the threshold level were considered not expressed. The validation of the RNA-seq results by qRT-PCR was performed as indicated in [11]. The employed primers are shown in Table S1.

### 2.4. Gene Ontology (GO) Analysis

The Gene Ontology (GO) terms were retrieved from Oleur061 [6] to be loaded in the Blast2GO software [20]. The GO term profile (combined graph) concerning all the GO term categories (molecular function, biological process, and cellular component) was performed. To make GO term profile comparisons between groups, the list of the genes expressed in at least one tissue was used as the reference gene set. The list of each tissue’s specific genes was compared with the reference set. GO enrichment was analyzed by a two-tailed Fisher’s exact test using a *p*-value threshold of 0.005.

### 2.5. Availability of Data

The raw RNA-seq datasets that supported the results of this study are deposited at the National Center for Biotechnology Information (NCBI) Gene Expression Omnibus (GEO). They are available under accession numbers GSE140648, GSM4176229, GSM4176230, GSM4176231, GSM4176232, GSM4176233, GSM4176234, GSM4176235, GSM4176236, GSM4176237, GSM4176238, GSM4176239, and GSM4176240 for Project PRJNA556567.

## 3. Results and Discussion

### 3.1. Transcriptome Profile Overview

**Figure 1:**
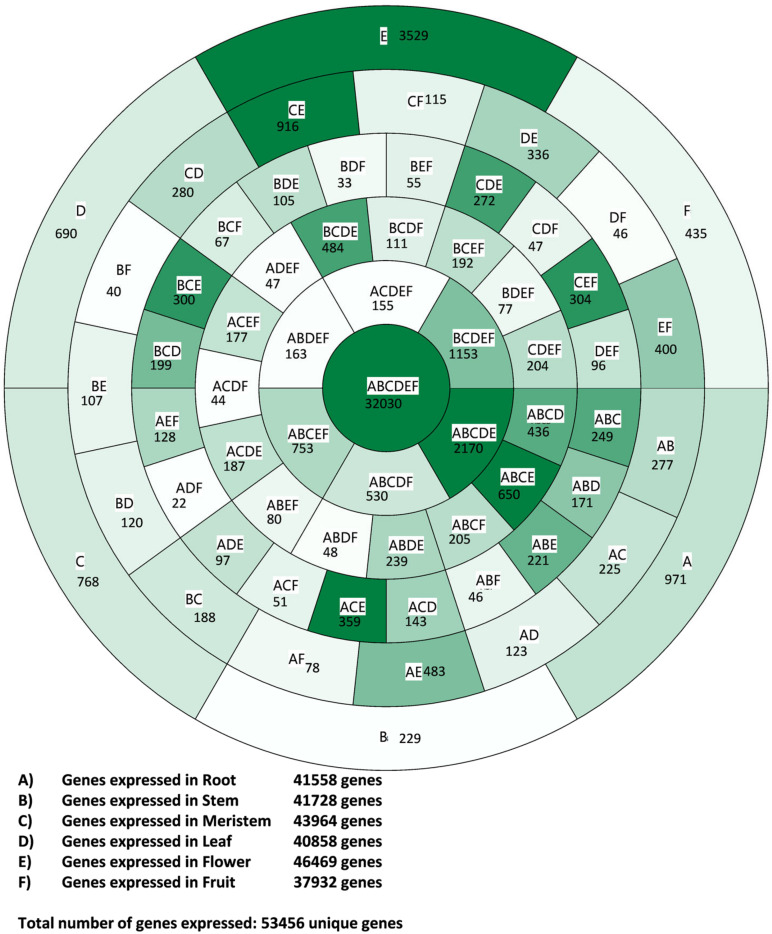
Venn diagram showing the gene expression profile of plant organs. Each compartment indicates the included organs and the number of genes expressed in it. A gene is considered expressed in an organ when its expression is > -2 log2 reads per kilobase per million mapped reads (RPKM) and “not expressed” when its expression is ≤ -2 log2 RPKM.

The expression of the genes in the roots, stems, meristems, leaves, mature flowers, and fruits (exocarp and mesocarp) from the “Picual” healthy and unstressed trees was analyzed by RNA-seq. The RNA-seq results were validated by qRT-PCR of 12 genes randomly selected among the different groups of genes analyzed below (Figure S1). The “Picual” genome contains 78,079 protein-coding genes, and from them 53,456 unique genes were found to be expressed at least in one of the six tested organs (Figure 1). The specific expression data at each organ for all the genes are provided in Table S2. The almost 25,000 unexpressed genes found in this RNA-seq could be expressed in other developmental stages or in response to biotic or abiotic stresses, or could simply be silenced. The high number of genes in the cultivated olive genome can be a consequence of two rounds of tetraploidy described in the evolution of the *Olea* genus. These events of tetraploidy have been dated around 57–63 million years ago (Mya) and 26–30 Mya by analyzing the wild olive genome [5], and around 62 Mya and 25 Mya by analyzing the “Picual” genome [6]. An additional very recent partial genome duplication event has been described in the cultivated olive genome [6]. Thus, there are many duplicated genes in the wild and even more in the cultivated olive genome. Some of them are not expressed at all, which is expected after genome duplication events.

The numbers of genes expressed in each organ did not significantly differ. They ranged from 46,469 in flowers to 37,932 in fruits, with 43,964 in meristems, 41,728 in stems, 41,558 in roots, and 40,858 in leaves (Figure 1). Some of these genes were exclusively expressed in one organ, whereas most were expressed in two organs or more, which produced a complex expression pattern that is portrayed in Figure 1. The full list of the different organ expression combinations of genes is provided Table S3. The list of the expressed genes with their GO terms can be found in Table S4.

### 3.2. Housekeeping Genes

**Figure 2:**
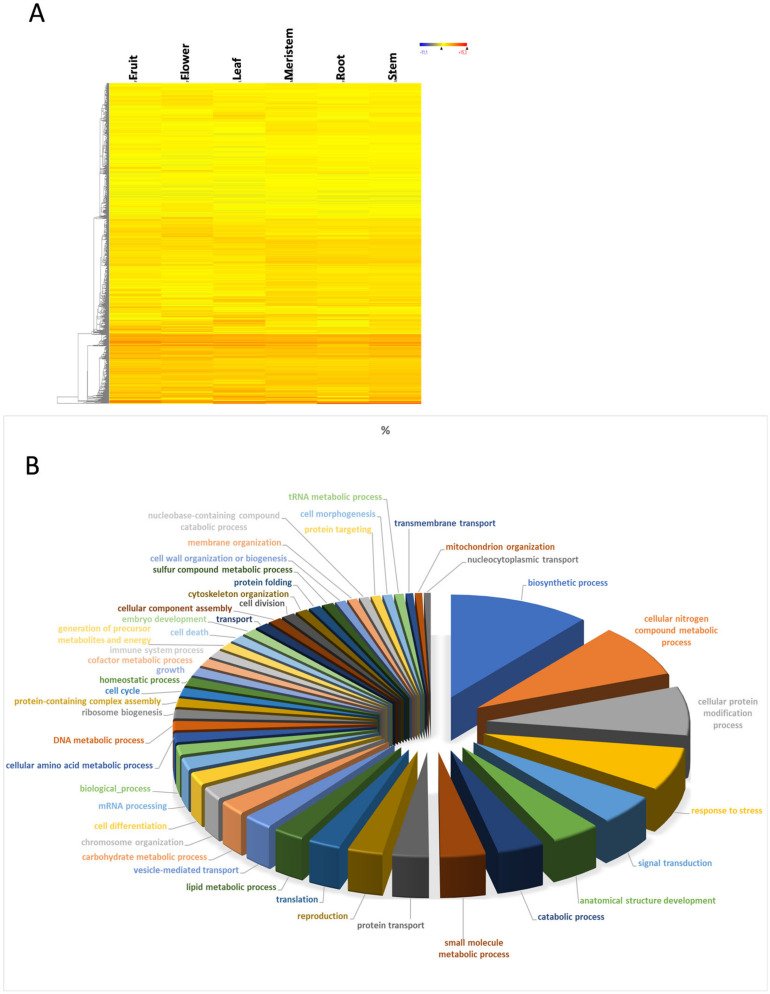
Olive housekeeping genes. Heat map of housekeeping genes (**A**) and the Gene Ontology (GO) term diagram of biological processes at level 5 (**B**).

The expression profiles in the six analyzed plant organs are shown in Figure 1. The fact that more than half the expressed genes (32,030) were expressed in all the tested plant organs suggests many putative housekeeping genes. However, many of these genes displayed very low expression levels in one organ or more, which means that their expression was organ- or tissue-dependent. To determine a more accurate number of housekeeping genes, a filter of high expression (log_2_ RPKM > 1) was set, and 19,575 genes were highly expressed in all the tested plant organs. In addition, most of these genes showed similar expression levels across all organs (Figure 2A). This set of unique genes could represent the cultivated olive housekeeping genes (Figure 2A and Table S5). Although the number of housekeeping genes in plants has not yet been established, 19,575 genes represent a high number compared to the 3804 estimated human housekeeping genes [21]. This high number of putative housekeeping genes could be due to the high number of paralogs present in the cultivated olive tree. In turn, such a high paralog content is likely to result from the two tetraploid events in the *Olea* genus [5,6], and the very recent partial genome duplication detected in the “Picual” genome [6].

The GO term descriptive analysis of these putative housekeeping genes showed that a large number of biological processes (BP) was represented (Figure 2B). Thus, the most represented BP are biosynthetic pathways, nitrogen compound metabolism, protein modification, response to stress, signal transduction, anatomical structure development, many metabolic and catabolic processes, protein biosynthesis, and transport. Despite containing fewer genes, other BP are essential for cell life, such as chromosome organization, mRNA processing, cell cycle, cell division, cytoskeleton organization, protein folding, and homeostatic process (Figure 2B and Table S6). The Kyoto Encyclopedia of Genes and Genomes (KEGG) pathway analysis also revealed a wide range of pathways, and most represented many genes involved in metabolism and biosynthetic pathways (Table S7). Therefore, the proposed housekeeping genes candidates found herein cover all the essential BP so that plant cells remain functional.

### 3.3. Fruit-Specific Genes

**Figure 3:**
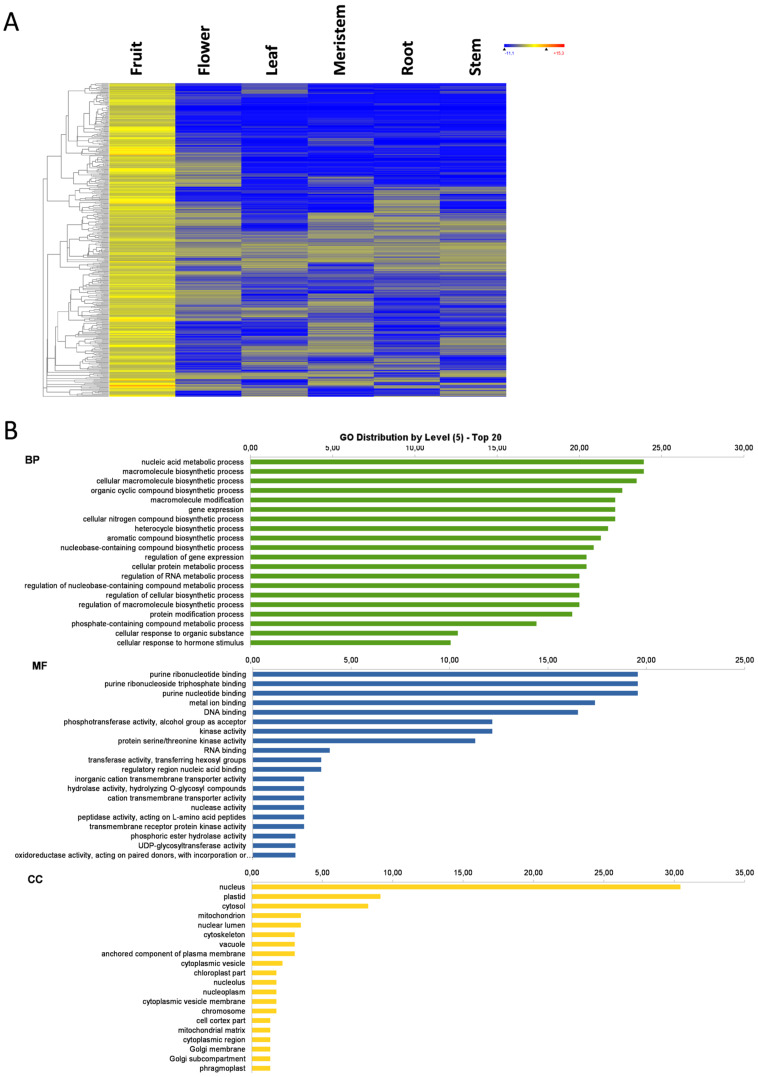

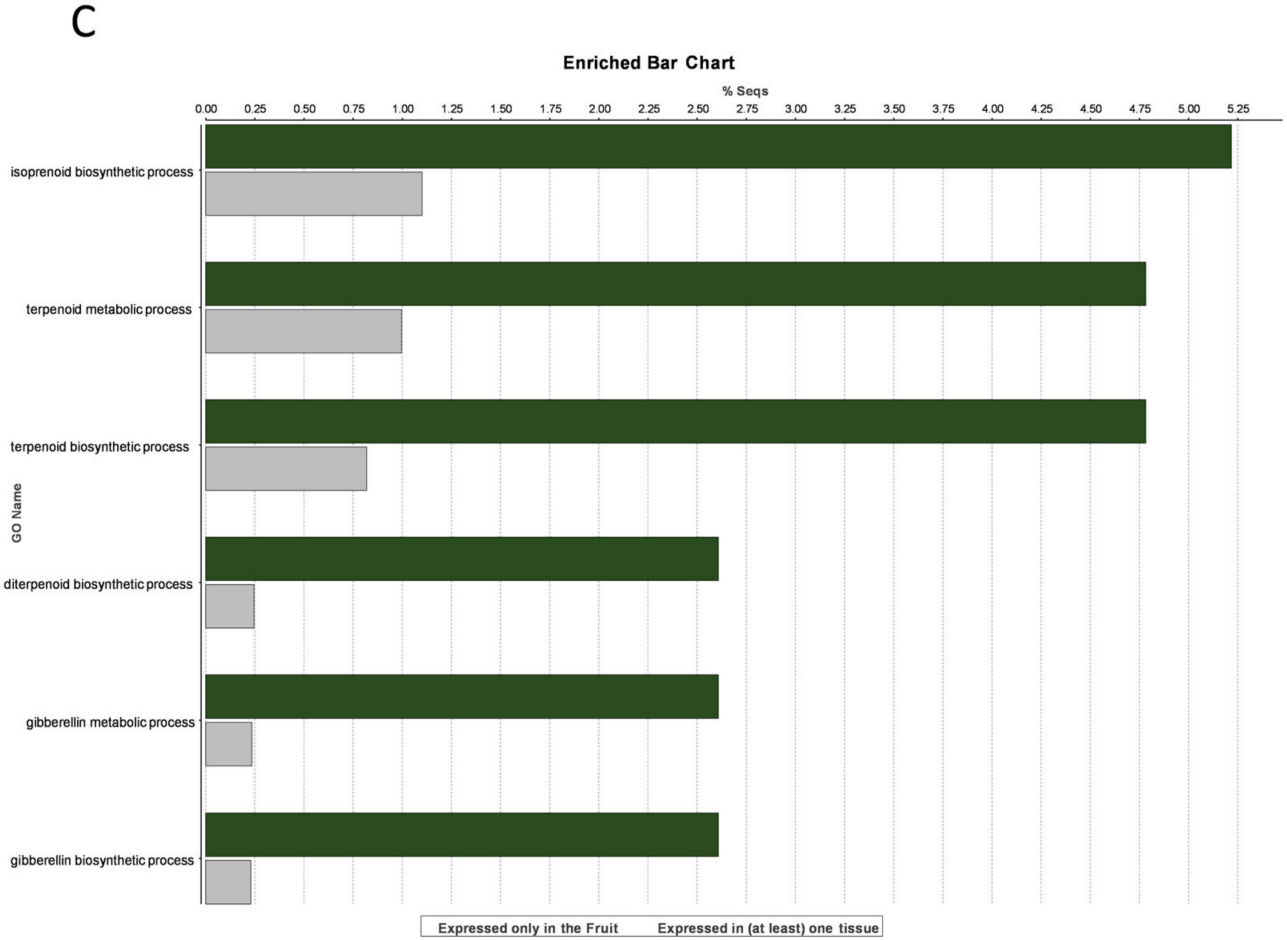
Fruit-specific genes. Heat map of fruit-specific genes (**A**); the GO term diagram of biological processes, molecular functions, and cellular components at level 5 (**B**); and the enriched biological process GO terms (**C**).

The 435 genes showing an expression above the basal level only in fruit and below the basal level in the other studied plant organs (Figure 1) were analyzed by GO term and KEGG ontology. The expression heat map of these genes and the GO distribution at level 5 are represented in Figure 3A,B, respectively. BP mostly included biosynthetic and metabolic processes, regulatory processes involved in gene expression, RNA metabolism, as well as organic and hormone responses. The molecular function (MF) was considerably high in purine-binding activities, DNA or RNA binding, and in kinase and transferase activities. The cellular component (CC) showed a fairly high nuclear localization, which suggests that many fruit-specific genes play a direct or indirect regulatory role (Figure 3B). The GO term-enriched analysis of the BP terms associated with the differentially expressed genes (DEGs) in fruit showed enrichment for the following six terms: isoprenoid biosynthetic process, terpenoid metabolic process, terpenoid biosynthetic process, diterpenoid biosynthetic process, gibberellin metabolic process, and gibberellin biosynthetic process (Figure 3C). The isoprenoid, terpenoid, and diterpenoid biosynthetic processes are likely involved in anthocyanin pigment biosynthesis, which is consistent with the phenolic compounds level change associated with the ripening stage of the analyzed samples, which were turning green to purple. Furthermore, the olive fruit samples reaching late maturity stages and before senescence, a process delayed by gibberellins [22,23]. In line with this, gibberellin biosynthesis and metabolism were also over-represented in the fruit-specific genes. This ripening stage was chosen to find genes related to maturity and olive oil production. In fact, the KEGG ontology analysis detected five fruit-specific genes involved in fatty acid biosynthesis and metabolism: two *LACS2*, one *FABZ*, and two *AAE*13 fruit-specific genes. *LACS2* genes encode long-chain acyl-CoA synthetase 2 enzymes involved in the activation of long-chain fatty acids for both synthesis of cellular lipids and degradation via beta-oxidation [24] and cuticle development [25]. The *AAE13* genes are also involved in fatty acid biosynthesis and encode Malonate--CoA ligases [26], but may also play a role in the detoxification of malonate [26]. *FABZ* gene is annotated by similarity to *Escherichia coli* genes and code for 3-hydroxyacyl-[acyl-carrier-protein] (ACP) dehydratase FabZ, which catalyzes the dehydration of short- and long-chain beta-hydroxyacyl-ACPs to produce unsaturated fatty acids [27]. In the future, an additional analysis of the whole set of genes in different development stages needs to be done to further characterize the gene expression of olive fruit.

### 3.4. Flower-Specific Genes

**Figure 4:**
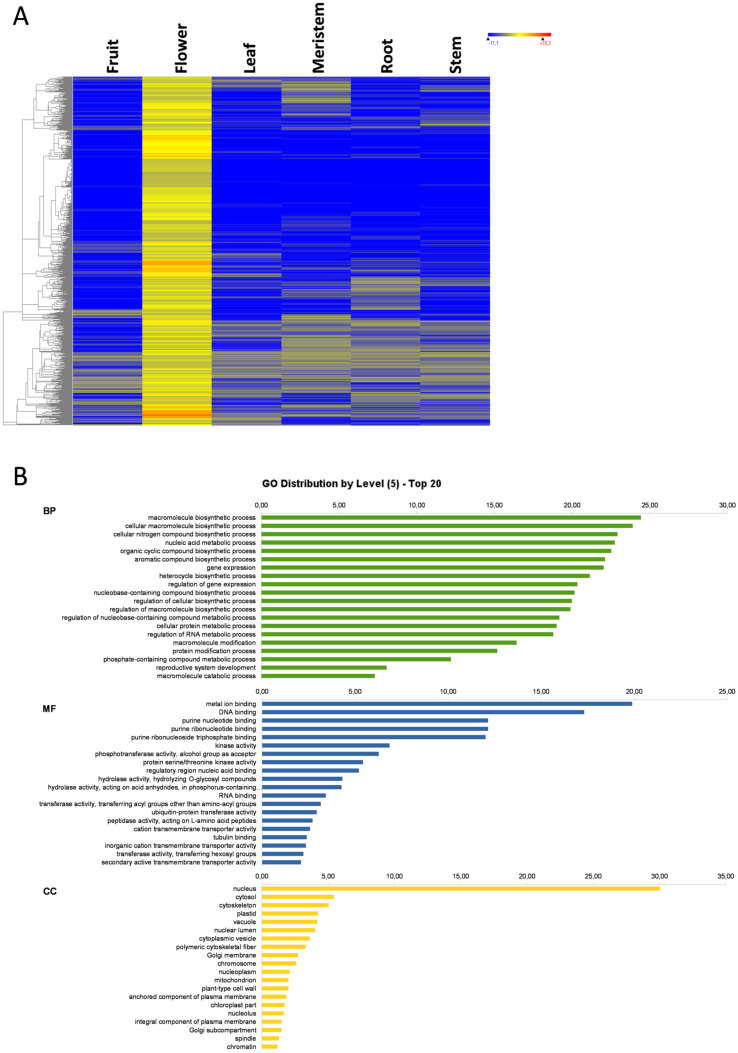

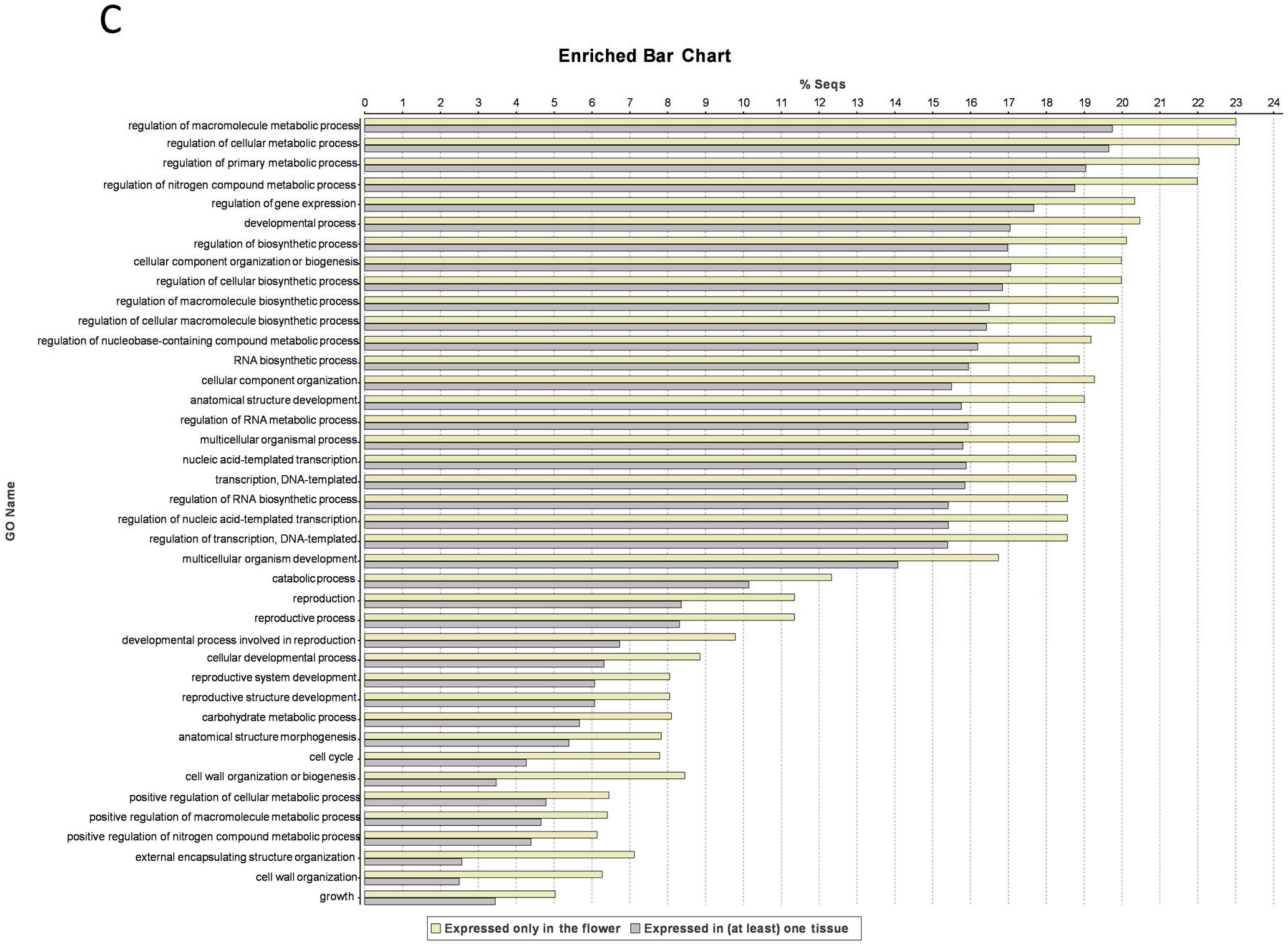
Flower-specific genes. Heat map of flower-specific genes (**A**); the GO term diagram of biological processes, molecular functions, and cellular components at level 5 (**B**); and the enriched biological process GO terms (**C**).

The largest number of organ-specific genes was found in flowers, as 3529 genes showed an expression above the basal level in flowers, and below the basal level in the other analyzed plant organs (Figure 1). The expression heat map of these flower-specific genes and the GO distribution at level 5 are represented in Figure 4A and B, respectively. In BP and KEGG terms, flower-specific genes were associated mostly with biosynthetic and metabolic processes, plant hormone signal transduction, plant-pathogen interaction, and regulatory processes involved in gene expression and RNA metabolism. As observed in fruit, genes were involved in pigment synthesis and in fatty acid biosynthesis and elongation. The reproductive system development process appeared among the top 20 more represented GO terms of the flower-specific genes. In this set of genes, 10 *CALS12*, 2 *CALS11*, and three *MS1* genes appeared as flower-specific. *MS1* genes encode for plant homeodomain (PHD) finger protein MALE STERILITY 1, are transcription factors that activate anther and post-meiotic pollen development and maturation, and control tapetal development [28,29]. *CALS* genes encode for callose synthases, which are involved in sporophytic and gametophytic development. They are required for forming the callose wall by separating tetraspores of the tetrad during pollen formation [30,31]. Most of the flower-specific genes were transcription factors, kinases involved in signal transduction, and auxin-response genes. According to the GO term enrichment analysis, most of the over-represented BP were related to gene regulation, metabolic and biosynthetic process regulation, developmental and growth processes, and reproduction (Figure 4C). Once again, the MF was considerably high in purine-binding activities, DNA or RNA binding, and kinase and transferase activities, but transmembrane activities and regulatory DNA-binding activities were also present. The most represented CC was nuclear localization. The BP, MF, and CC GO terms suggest that, as with fruit, most of the flower-specific genes play a putative regulatory role (Figure 4B).

### 3.5. Leaf-Specific Genes

**Figure 5:**
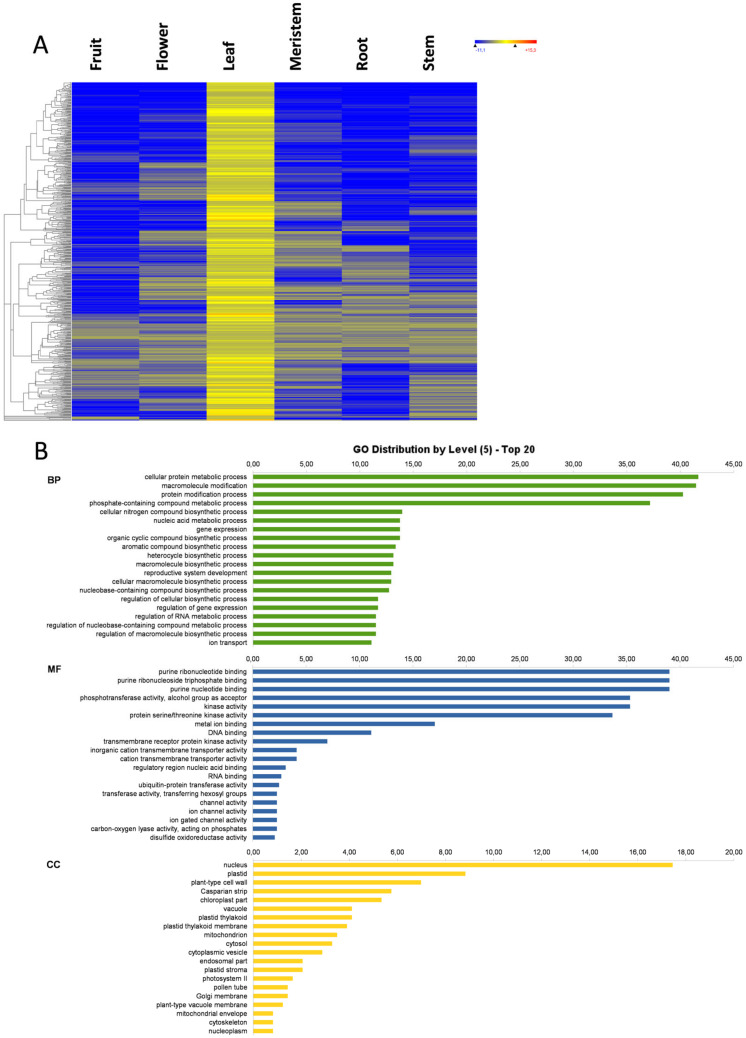

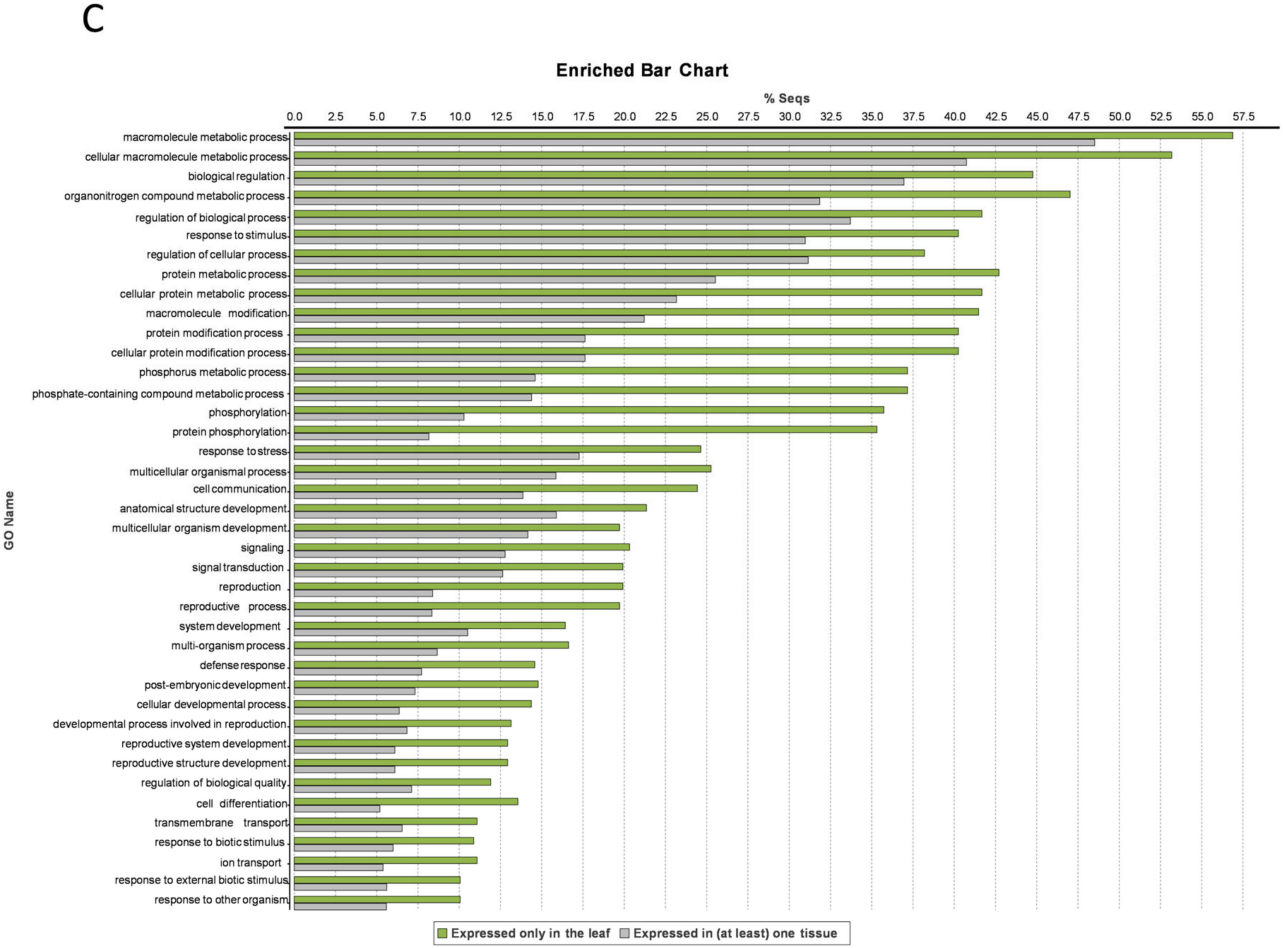
Leaf-specific genes. Heat map of leaf-specific genes (**A**); the GO term diagram of biological processes, molecular functions, and cellular components at level 5 (**B**); and the enriched biological process GO terms (**C**).

In leaves, 690 specific genes were found (Figure 1), whose expression heat map and the GO term distribution at level 5 are represented in Figure 5A and B, respectively. As in the previous organs, BP included mostly biosynthetic and metabolic processes, regulatory processes involved in gene expression, and RNA metabolism processes. However in flowers, the reproductive system development appeared as highly enriched GO terms (Figure 5C), and also in KEGG terms among the leaf-specific genes (Table S7). The ion transport process appeared among the top 20 most represented GO terms of leaves. The most represented MF was, once again, purine binding, DNA or RNA binding, and kinase and transferase activities, although many metal ion binding and ion channels were also found, which suggests a large specific ion transport system, which is consistent with the BP terms result. Although the CC GO terms were also numerous at nuclear localization, their number was not as high as in other organs. Instead, a large number of CC terms were plastids, thylakoids, photosystem II, and photosynthesis-related GO terms. The BP, MF, and CC GO terms suggest that, as in the previous organ-specific genes, a number of leaf-specific genes may play a regulatory role, but many others are involved directly in plastid and photosynthesis activities (Figure 5B). It is noteworthy that pigments, flavonoids, and polyphenols accumulate in olive leaves [32]. This is important because the flavonoids present in leaves such oleuropein possess proven anticancer activity [33]. Among the leaf-specific genes, a *DFRA* gene was found that encodes for dihydroflavonol 4-reductase, involved in flavonoid biosynthesis. A *LCY1* gene, which encodes for a chloroplastic lycopene beta cyclase involved in β-carotene and β-zeacarotene biosynthesis, was also detected in this set of leaf-specific genes. There was also a set of leaf-specific genes implicated in the response to external stimulus. In this case, two genes involved in leaf circadian rhythm were included in the leaf-specific genes set. One of them is an *EARLY FLOWERING 3* (*ELF3*) gene that encodes a circadian clock-regulated nuclear protein in *Arabidopsis* [34], and a *CRY1* gene that encodes a cryptochrome/DNA photolyase, a FAD-binding domain protein. Cryptochromes are blue-light photoreceptors involved in the circadian clock for plants and animals [35,36]. In addition, reproduction, development, and transmembrane transport were over-represented. Therefore, leaf-specific genes are partially involved in gene regulation and partly located in structures and involved in photosynthesis-related activities, such as plastid development, thylakoid structure, phosphorylation and transmembrane transport, as well as pigment and flavonoid biosynthesis and circadian rhythm.

### 3.6. Meristem-Specific Genes

**Figure 6:**
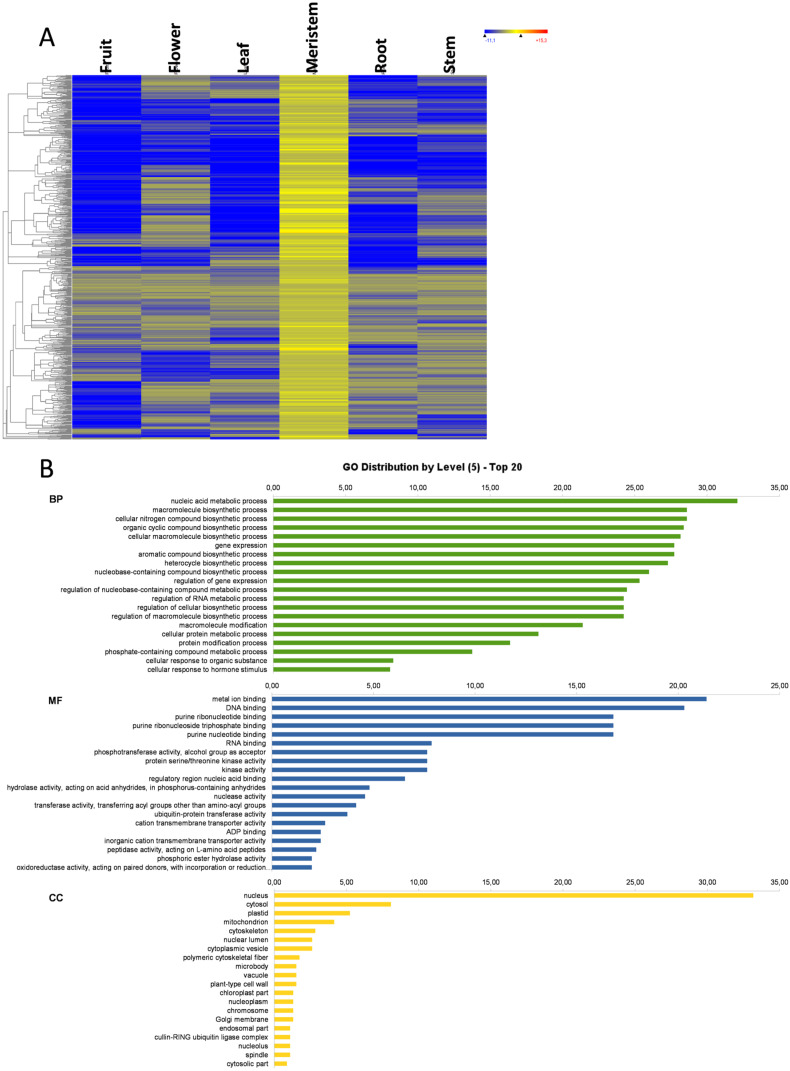

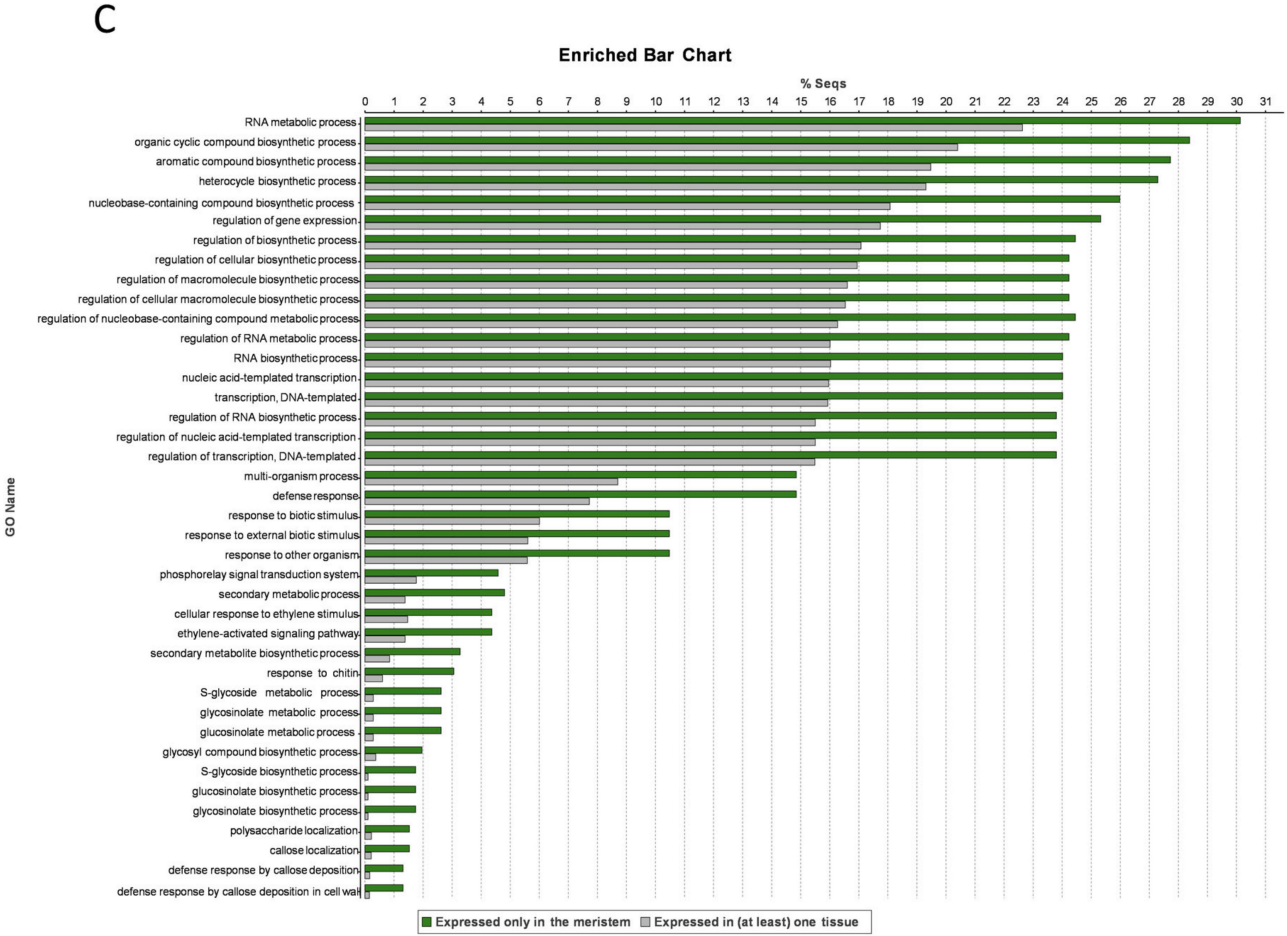
Meristem-specific genes. Heat map of meristem-specific genes (**A**); the GO term diagram of biological processes, molecular functions, and cellular components at level 5 (**B**); and the enriched biological process GO terms (**C**).

The selection of meristem-specific genes produced a set of 768 genes (Figure 1). The heat map of these genes and the GO distribution at level 5 are represented in Figure 6A and B, respectively. The meristem is quite a undifferentiated tissue, and thus it is not surprising that the GO and KEGG ontology show a wide range of processes, including biosynthetic and metabolic processes, gene expression and its regulation, RNA metabolism, and protein modification. Additionally, among the top 20 most represented GO terms, cellular response to organic substance and to hormone stimulus was found. The most represented MF GO term was metal ion binding, followed by DNA or RNA binding, purine binding activities, kinase and phosphotransferase activities, as well as several transferase activities. In addition, hydrolase, peptidase, transmembrane transport, and oxidoreductase activities were represented. In meristems, the specific genes’ GO terms regarding CC nuclear localization were by far the largest group. These GO terms suggest that, as in previous organ-specific genes, most of the meristem-specific genes may play a regulatory role by controlling a wide range of activities required for cell metabolism (Figure 6B). The GO term-enriched analysis of meristem-specific genes’ BP resulted in a large number of biosynthetic and metabolic processes, regulation of gene expression, and response to external stimulus and to ethylene. In addition, some GO terms related to defense against pathogens as a response to chitin or callose deposition were present among meristem-specific genes (Figure 6C). Therefore, meristem-specific genes are involved mostly in the gene regulation of various cell processes that govern the necessary response to external and internal stimuli to proliferate. Additionally, a set of meristem-specific genes for defense against biotic stresses was also found. 

### 3.7. Root-Specific Genes

**Figure 7:**
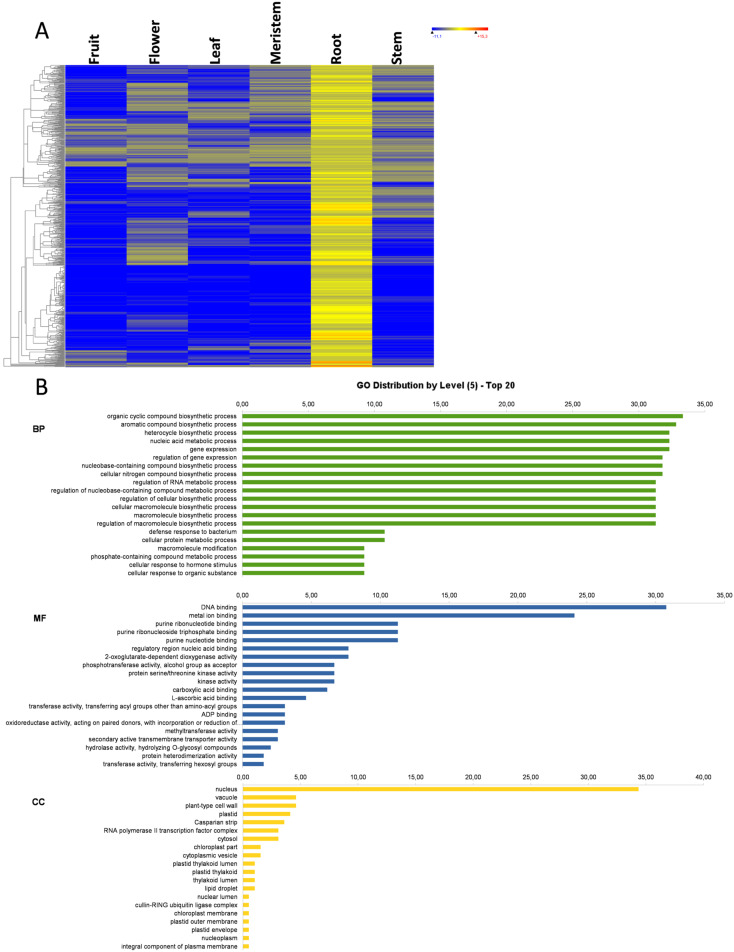

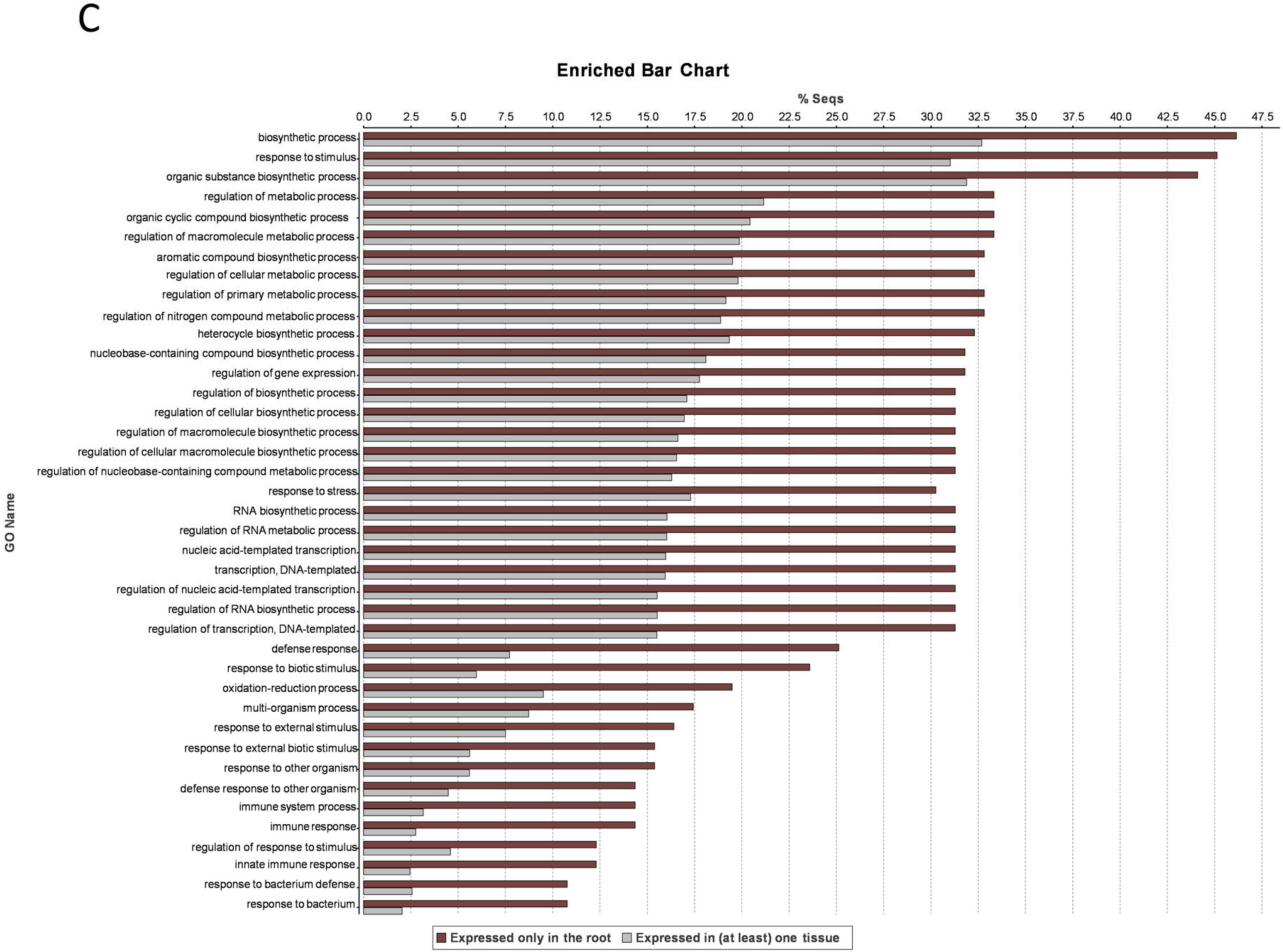
Root-specific genes. Heat map of root-specific genes (**A**); the GO term diagram of biological processes, molecular functions, and cellular components at level 5 (**B**); and the enriched biological process GO terms (**C**).

A total of 971 root-specific genes (Figure 1) were found. The expression heat map of these genes and the GO distribution at level 5 are represented in Figure 7A and B, respectively. Although the BP and KEGG terms were quite similar to the other organ-specific genes, including mostly biosynthetic and metabolic processes, gene expression and its regulation, RNA metabolism, and the cellular response to organic substance and hormone stimuli (Figure 7B and Table S7), defense genes were highly enriched (Figure 7C). In fact, the GO term enrichment analysis of root-specific genes BP showed not only a significantly large number of biosynthetic and metabolic processes, and regulation of gene expression, but also many defense response-related GO terms, with many being associated with immune response and response to bacterium (Figure 7C). The most represented MF GO terms were DNA and regulatory region nucleic acid binding, as well as metal ion binding, followed by purine binding activities, kinase and phosphotransferase activities, several transferase activities, carboxylic and L-ascorbic acid-binding activities, secondary active transmembrane transporter activities, and oxidoreductase and hydrolase activities. The most represented CC GO term was nuclear localization. This GO term analysis suggests that, as in previous organ-specific genes, most root-specific genes may play a regulatory role in controlling a wide range of activities required for cell metabolism (Figure 7B). Roots are exposed particularly to rhizosphere microorganisms, some of which penetrate roots and become endophytes [37], which may explain why the root has its own set of defense genes. Of the defense genes found among the root-specific genes, 15 contained a START-like domain (StAR-related lipid-transfer) present in the signal-transduction proteins [38]. Ten of them were genes that encode major latex proteins (MLP-like): three *MLP146*, three *MLP28*, two *MLP423*, one *MLP31*, and one *MLP*43. These are defense genes, and MLP43 confers drought tolerance in *Arabidopsis* [39]. Additionally, 16 of the root-specific genes encode AT-hook motif nuclear-localized protein 20 (plant and prokaryotes conserved (PPC) domain). These genes encode transcription factors that negatively regulate plant innate immunity (PTI) to pathogens [40], which suggests that the roots of healthy plants at least partially repress PTI, but also partially induce root-specific defense genes, such as *MLP* genes and the disease-resistance *RPP8* gene.

### 3.8. Stem-Specific Genes

**Figure 8:**
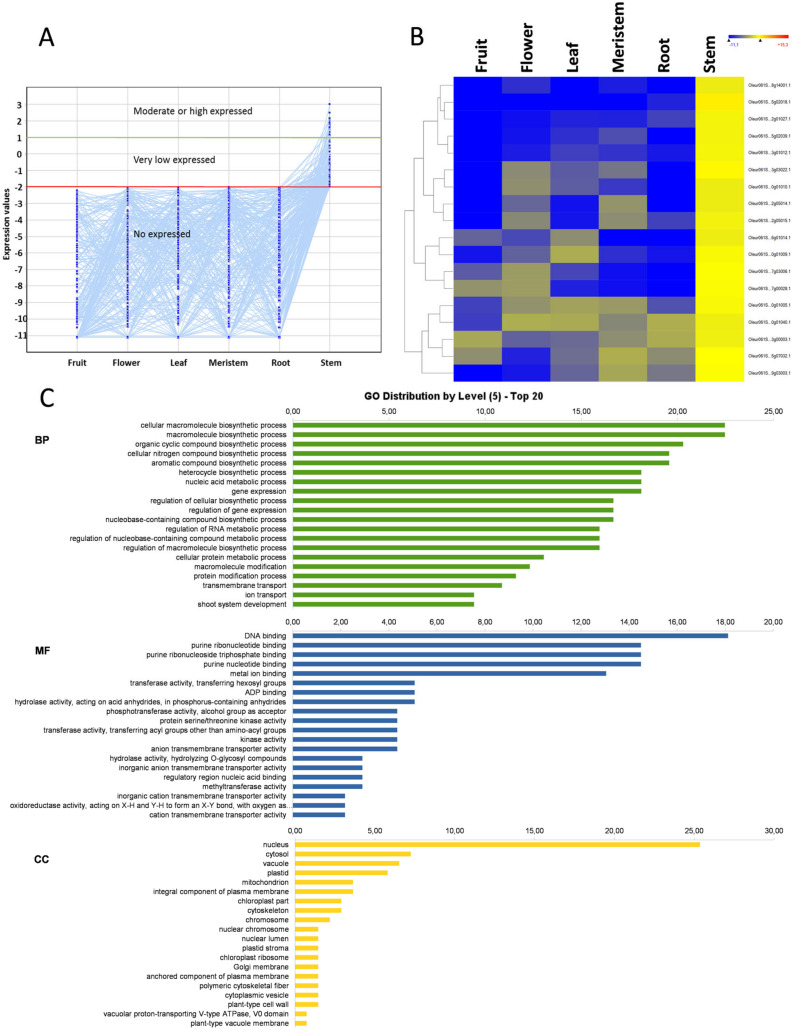
Stem-specific genes. Line graph of root-specific genes (**A**); the heat map of the moderate or high expressed root-specific genes (**B**); and the GO-term diagram of biological processes, molecular functions, and cellular components at level 5 (**C**).

The stem had the fewest organ-specific genes represented by only 229 genes (Figure 1). In addition, the expression level of most of these genes was very low, only slightly above the basal-level threshold (Figure 8A). In fact, only 18 genes showed an expression level of log_2_ RPKM > 1, an 8-fold change above the basal level (Figure 8B). Such a small number of genes (229) did not cluster in any statistically significant GO term after the GO enrichment analysis. However, the GO term at level 5 and the KEGG term distribution results were quite similar to the other organ-specific genes (Figure 8C and Table S7). As most stem-specific genes had a very low expression level, we focused on the 18 genes with at least a moderate expression level. Fifteen of them were functionally annotated and comprised two serine-threonine kinases, four oxide-reductases, six enzymes of different metabolic pathways, two transcription factors, and one gene involved in phototropism. This last one was a *PKS1* gene that encodes a PHYTOCHROME KINASE SUBSTRATE 1 protein involved in light signaling in *Arabidopsis* [41]. In fact, this protein is required for phototropism [42], and also for gravitropism in roots [43]. Therefore, this protein is a candidate to play a role in controlling the stem phototropism and/or anti-gravitropism. Regarding transcription factors, one of them was a dehydration-responsive element-binding protein 1F (*DREB1F*) gene, which is involved in freezing tolerance and cold acclimation [44], whereas the other was an *MYB113*, which is likely implicated in regulating the development process or stress response. 

## 4. Conclusions

The RNA-seq transcriptomic analysis of the fruit, flower, leaf, meristem, root, and stem samples from adult healthy plants revealed the expression of 53,456 unique genes, which means that there are nearly 25,000 genes in the genome that might be expressed in other developmental stages in response to biotic or abiotic stresses, or simply silenced. A full complex gene expression pattern is provided. Of these 53,456 unique genes, many (19,575) possible housekeeping genes were found as they were expressed at moderate or high levels in the six analyzed plant organs. Additionally, organ-specific genes were defined. Flowers showed a significantly large number of specific genes (3529), many of which were related to reproduction, including three male sterility *MS1* genes and 12 sporophytic and gametophytic development *CALS* genes. It can be concluded that the genetic and metabolic regulatory processes that control floral development require the involvement of at least 3529 of the genes together, which are specifically expressed in flowers. In fruit, there were fewer specific genes (435), with some genes involved in fatty-acid biosynthesis and also in pigmentation. In general, many of these organ-specific genes are involved in regulatory activities and show nuclear protein localization, except for leaves, in which the coded proteins of many genes were located at the plastid, which also happens in stems to a lesser extent. In leaves, 690 tissue-specific genes were found and included some genes involved in the biosynthesis of flavonoids and carotenes with a marked presence in olive leaves, as well as genes involved in the circadian rhythm. Very few stem-specific genes with a moderate or high expression were found, including a *PKS1* gene that could play a role in controlling stem phototropism and/or anti-gravitropism. Pathogen defense pathways were very well represented in roots. Interestingly, the genes that negatively regulated the PTI were specifically expressed in roots, which suggests that the roots of healthy plants had at least partially repressed the PTI, but had also partially induced root-specific defense genes. Meristems presented a wide range of processes in the 768 tissue-specific genes, which is consistent with being a very undifferentiated tissue. A full list of all the gene profiles is provided, together with the expression level data for all the genes. These data might be an extremely useful tool for seeking out future breeding genetic markers.

## Supplementary Materials

The following are available online at https://www.mdpi.com/2073-4425/11/5/544/s1. Figure S1: RNA-seq validation by qRT-PCR. Table S1: Primers used in the qRT-PCR assays. Table S2: Expression data of all the fruit, flower, leaf, meristem, root, and stem genes. Table S3: List of gene IDs comprised in the compartments of the Venn diagram shown in Figure 1. Table S4: List of gene IDs with their GO terms and molecular function, biological process, and cellular component. Table S5: List of gene IDs of housekeeping genes. Table S6: Full biological process GO-term list of housekeeping genes. Table S7: KEGG terms of housekeeping and fruit-, flower-, leaf-, meristem-, root-, and stem-specific genes.
