# Supplementary material for: Gene Expression Pattern in Olive Tree Organs (Olea europaea L.)
Source: Genes (Basel). 2020 May 12;11(5):544. doi: 10.3390/genes11050544 (PMC7291012; doi:10.3390/genes11050544)
Supplement: Supplementary file 1 [file genes-11-00544-s001.zip › Supplemental Table S1.docx]

**Table S1.** Primers for qRT-PCR.

|  | **Primer name** | **Primer sequence** | **Amplicon size (bp)** |
| --- | --- | --- | --- |
| Oleur061Scf8354g00019.1 | 8354g00019F | GGCATTATTGTCATTTAGGTTTCCTTAGTTGC | 164 |
|  | 8354g00019R | AGTAGCAAAATTTTGAAAGTTCGGGATGG |  |
| Oleur061Scf3725g11030.1 | 3725g11030F | AAAACATGGATTAATCATGTGGCTGCAC | 164 |
|  | 3725g11030R | TGAGATAAACGAAAAAGGAAGCAAGACACTG |  |
| Oleur061Scf6083g02012.1 | 6083g02012F | TGCAAGTTATTGTTTTCGGCGAAGTAGTG | 225 |
|  | 6083g02012R | AAAATGTGATAAAATAATCTGCTCCAGAACACG |  |
| Oleur061Scf0802g01015.1 | 0802g01015F | ATTTGCTTCCAAACTGTCGGTTCAGTG | 196 |
|  | 0802g01015R | TGTTGCAAAATCTGAGTTAGATTTTCGTTGG |  |
| Oleur061Scf1658g01009.1 | 1658g01009F | ATTTCATGCATTACTGCTAGTAAGGTTTTTCTAGG | 188 |
|  | 1658g01009R | TTTAACGGTAGCTACAATGTCGAATTACAAAGAG |  |
| Oleur061Scf3259g05001.1 | 3259g05001F | TCGTGCAAACCCTCAAAGAACAATAACC | 186 |
|  | 3259g05001R | CTTATCGCGCACTTGGCCTACATACTC |  |
| Oleur061Scf0625g00032.1 | 0625g00032F | TCTCCCTCCGTATAGCTTCACTAAATCATCTC | 181 |
|  | 0625g00032R | TGAACTAGAGTTGAGAAAATCACGAAGAAGAACC |  |
| Oleur061Scf3080g03011.1 | 3080g03011F | AGATTCTCTCTAAAGGAACCACCAGACTCG | 158 |
|  | 3080g03011R | GAACGCGAACATGAGTAGCTTTAAGTTGG |  |
| Oleur061Scf2029g00022.1 | 2029g00022F | TGACGCTTTGCTGCAACAACAGGAG | 177 |
|  | 2029g00022R | AAAGACGGGTACGCTTACACAATGTAGTCTC |  |
| Oleur061Scf6430g01008.1 | 6430g01008F | CATGTTCCCAGTGTCGCTATAAATAGATTAAGAG | 160 |
|  | 6430g01008R | ATTTCAATTTGGGCAGTTAGTTTACCTGTAAGAC |  |
| Oleur061Scf0925g02018.1 | 0925g02018F | TTATGTAATTCACTTAATTTGGATGCTTGGTGC | 189 |
|  | 0925g02018R | CCCAAAGCATCTACTGCACAAACATATCC |  |
| Oleur061Scf1163g03022.1 | 1163g03022F | GCCAATCAAGAATTTAGCTCATGCTTCG | 205 |
|  | 1163g03022R | GCAAGACAGAGTAATACAATTTGGTACAGATATGG |  |
