# Supplementary figures and images for: Gene Expression Pattern in Olive Tree Organs (Olea europaea L.)
Source: Genes (Basel). 2020 May 12;11(5):544. doi: 10.3390/genes11050544 (PMC7291012; doi:10.3390/genes11050544)

**Figure S1.** RNA-seq validation by qRT-PCR.


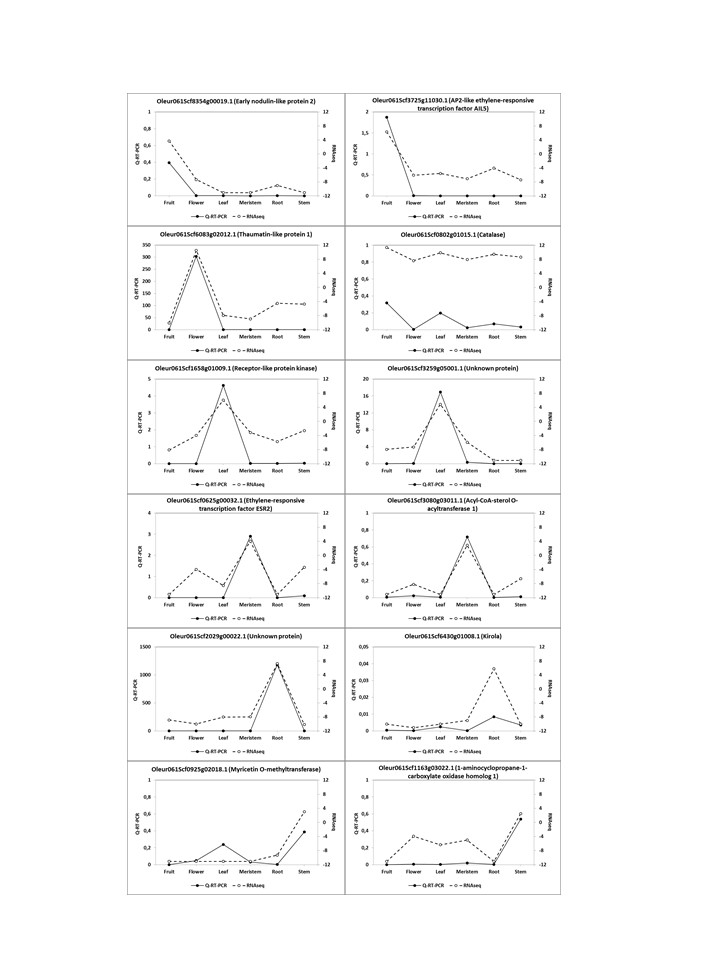

Supplement: Supplementary file 1 [file genes-11-00544-s001.zip › Supplementary Figure S1.docx]
